# Novel approaches for HTLV-1 therapy: innovative applications of CRISPR-Cas9

**DOI:** 10.1590/S1678-9946202466048

**Published:** 2024-08-26

**Authors:** Wilson Domingues, Victor Ângelo Folgosi, Sabri Saeed Sanabani, Pedro Domingos Leite, Tatiane Assone, Jorge Casseb

**Affiliations:** 1 Universidade de São Paulo, Faculdade de Medicina, Divisão de Dermatologia, Laboratório de Investigação Médica LIM-56, São Paulo, São Paulo, Brazil

**Keywords:** HTLV-1, Gene editing, CRISPR-Cas9, TAX, HBZ

## Abstract

The human T-cell lymphotropic virus type 1 (HTLV-1) is a single-stranded positive-sense RNA virus that belongs to the *Retroviridae* family, genus Deltaretro, and infects approximately five to 10 million people worldwide. Although a significant number of individuals living with HTLV-1 remain asymptomatic throughout their lives, some develop one or more severe clinical conditions, such as HTLV-1-associated myelopathy/tropical spastic paraparesis (HAM/TSP), a progressive and debilitating disease, and/or a subtype of non-Hodgkin’s lymphoma with a more threatening course known as adult T-cell leukemia/lymphoma (ATLL). Moreover, current therapeutic options are limited and focus primarily on treating symptoms and controlling viral latency. CRISPR-Cas9 gene editing is proposed as a promising tool to address the intricate links associated with HTLV-1. By targeting or silencing key genes during initial infection and dysregulating immune signaling pathways, CRISPR-Cas9 offers potential intervention opportunities. In this review, we address the therapeutic potential of CRISPR-Cas9 gene editing, as well as examine the primary mechanisms involved in editing potential target genes and discuss the existing evidence in the current scientific literature.

## INTRODUCTION

The human T-lymphotropic virus type 1 (HTLV-1), a positive-sense single-stranded RNA retrovirus that belongs to the *Retroviridae* family, genus Deltaretro, infects approximately five to 10 million people worldwide^1^. HTLV-1 is mainly transmitted by sexual contact, blood transfusion, and from mother to child via breastfeeding^1^. HTLV-1 exhibits higher prevalence rates in specific regions, such as Japan, the Caribbean, and sub-Saharan Africa^1^.While most HTLV-1 infections remain asymptomatic, a small percentage of carriers (around 3–5%) may develop debilitating clinical conditions with uncertain prognosis. Those include HTLV-1-associated myelopathy/tropical spastic paraparesis (HAM/TSP), and/or adult T-cell leukemia/lymphoma (ATLL)^2^.

HAM/TSP is characterized by progressive neurological symptoms, such as weakness and stiffness in the legs, urinary dysfunction, and sensory abnormalities^1^. The onset of these symptoms typically occurs during the fourth or fifth decade of life, with a higher incidence in women^3^. The pathogenesis of HAM/TSP involves a chronic inflammatory response in the central nervous system, predominantly affecting the thoracic spinal cord^4^. Although the exact mechanisms underlying disease progression are not fully understood, it is believed that the disease progression can be categorized into three main courses: rapid, mild, and slow, which involve both viral and host factors^5^. Furthermore, it was suggested by Haziot *et al.* ^6^ that HTLV-1-infected patients, initially classified as asymptomatic, exhibited previously unrelated neurological symptoms. These patients were then reclassified as carriers of an intermediate syndrome (IS), indicating an intermediary clinical form between the asymptomatic phase and HAM/TSP. Symptoms associated with IS included neurological changes, visual disturbances, oral conditions, skin lesions, bladder disturbances, and rheumatological symptoms. Additionally, the HTLV-1 proviral load, bladder disturbances, and rheumatological symptoms were identified as independently associated factors with IS.

On the other hand, ATLL represents a malignancy originating from mature T-cells. This condition is closely associated with profound immunosuppression, fostering aggressive tumor growth and systemic manifestations such as fever, weight loss, and lymphadenopathy^7^. Despite intensive chemotherapy regimens, the prognosis for ATLL remains dismal, with most patients succumbing to the disease not long after diagnosis, from 5.5 to 13 months^7^. Furthermore, ATLL can be classified into four subtypes according to the Shimoyama criteria, wherein the acute and lymphoma subtypes are notably aggressive, whereas chronic and smoldering ATLL tend to exhibit a more indolent disease course^8^.

Numerous ongoing research endeavors aim to identify a molecular entity crucial for determining the disease unfavorable prognosis. Despite remarkable progress, significant questions remain regarding the impacted pathways of immune signaling, as well as the insufficiency of genuinely efficacious therapeutic interventions^9^. One of the most prominent strategies involves the use of corticosteroids. However, while corticosteroids have shown effectiveness in alleviating symptoms and controlling viral latency, their overall impact on the disease progression and patient outcomes remains uncertain and subject to debate^10^.

At the same time, several studies have been conducted to explore the intricate molecular mechanisms of HTLV-1-related diseases, particularly ATLL. Furthermore, other studies have revealed the dysregulation of various signaling pathways ranging from T-cell activation to survival, while highlighting the central role of viral factors in modulating the host immune response and promoting oncogenesis^11^. Advancements in understanding the genetic and epigenetic alterations associated with HTLV-1 infection and ATLL development are fostering hope that precision medicine approaches can be developed, tailored to individual patients’ molecular profiles^12^.

Research suggests that the TAX protein, a trans-activating protein that regulates viral gene expression and also modulates cellular signaling pathways to enhance T-cell proliferation and cell survival, plays a pivotal role in dampening the transcriptional activation of IFN-β, a key antiviral cytokine, triggered by the overexpression of STING (Stimulator of Interferon Genes)^13^. TAX exerts a negative regulatory effect on the stimulated production of IFN-β, mediated by various molecules such as poly(dA:dT), interferon-stimulatory DNA (ISD), or cyclic GMP-AMP synthase (cGAS)^13^. These molecules are integral components in the activation of the cGAS/STING pathway, crucial for sensing cytosolic DNA and initiating innate immune responses^14^. The cGAS/STING pathway plays a pivotal role in orchestrating the elimination of pro-inflammatory DNA and triggering cellular autophagy, thereby contributing, or in this case failing to contribute, to the host’s defense against viral infections^15^.

**Figure 1:**
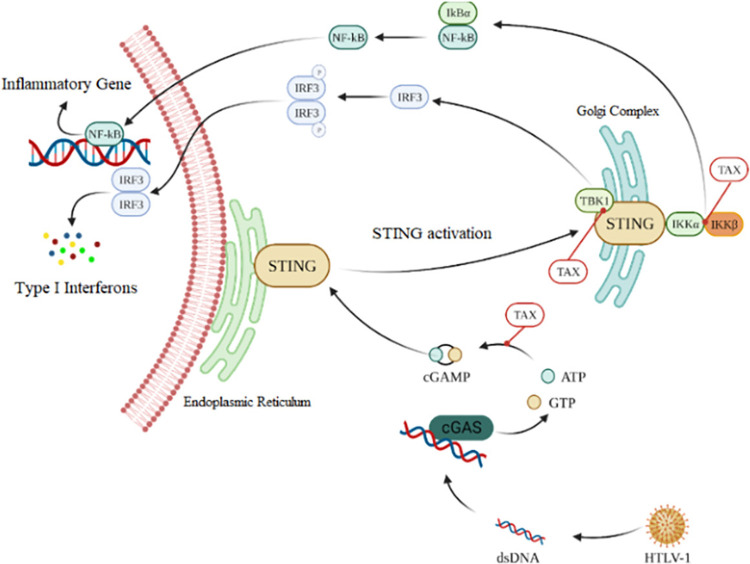
Activation and inhibition of cGAS/STING signaling during HTLV-1 infection.

Due to the dampened activation of IFN-β and subsequent impairment of the Beclin 1 autophagy complex, accumulated pro-inflammatory DNA perpetuates the sustained activation of the inhibitor of κB kinase (IKK) complex^16^. This persistent signaling leads to continuous nuclear factor kappa B (NF-kB) activation, triggering prolonged inflammatory processes^16^. Ultimately, this cascade directly contributes to conditions such as HAM/TSP and promotes cellular senescence associated with ATLL^17^ (Figure 1).

The proviral genome of HTLV-1 is complex and contains several elements that play crucial roles in the infection pathogenesis. In addition to TAX, another critical element is the HTLV-1 basic leucine zipper factor (HBZ), an antisense transcript that is approximately 9 kb in size^18^. The expression of HBZ is meticulously regulated by promoters located at the 5’ and 3’ ends of the virus’ long terminal repeats (LTRs). This finely tuned regulation allows HBZ to be consistently expressed in leukemic cells and to perform functions of critical importance in the pathogenesis of ATLL^19^.

**Figure 2:**
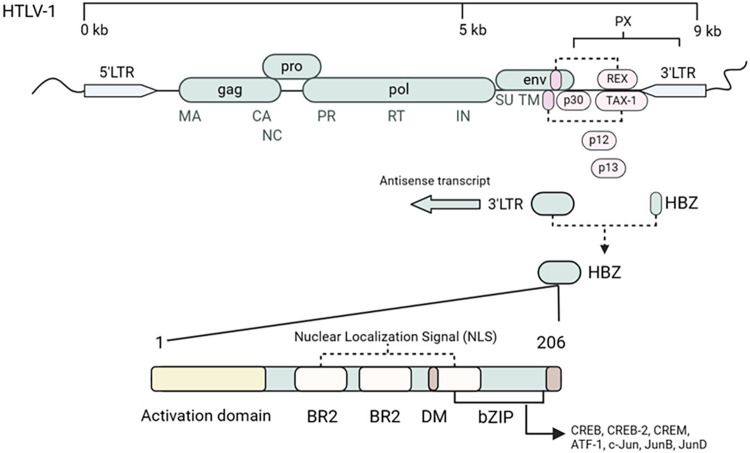
Expression and activities of HBZ RNA and protein. Representative schematic of the HTLV-1 genome and its interactions with host factors. The essential genes include gag, pro, pol, env, TAX-1, and HBZ. These genes encode essential subproteins for viral assembly and the replication cycle are encoded. The gag gene codes for structural proteins such as the capsid (CA), the matrix (MA), and the nucleocapsid (NC) are encoded. The pro gene codes for proteins that are required for processing viral polyprotein. The pol gene encodes essential enzymes for viral replication, such as reverse transcriptase (RT) and integrase (IN). The viral envelope proteins (SU-surface and TM-transmembrane) are encoded by the env gene. Highlighted interactions also include the negative-sense transcription of HBZ and its relationship with transcription factors such as c-Jun, JunB, and CREB2, which influence host transcription factors and the immune response.

Unlike the TAX protein, whose expression varies, HBZ is consistently expressed in ATL-affected cells, making it a pivotal component of the virus life cycle^20^. HBZ is strongly linked to the proviral load and significantly contributes to the proliferation of infected T cells. *In vitro* and animal model investigations have revealed that HBZ interacts with multiple host factors, including c-Jun, JunB, JunD, CREB2, and CREB, exerting a negative impact on the transcription of both viral and cellular genes^21^ (Figure 2).

Delving into the intricate role of HBZ within HTLV-1 infection stands as a pivotal endeavor for advancing novel therapeutic interventions^22^. The manipulation of HBZ gene dynamics and its interplay with cellular elements presents a promising path to disrupt ATLL advancement, countering the deleterious effects propagated by TAX, and vice versa^22^. Studies into these genes functions could therefore shed light on the underlying mechanisms of the pathogenesis of various HTLV-1-related diseases. Such an approach could significantly impair infected cells proliferation and viability, thereby dampening the immunomodulatory effects and ultimately reducing the burden of associated diseases^19^.

### CRISPR-Cas9 systems

The CRISPR-Cas9 system, a revolutionary discovery in molecular biology, represents an adaptive defense mechanism found in bacteria and archaea^23^. Its primary function is to protect organisms against invasions of exogenous nucleic acids, such as phages or plasmids^24^. By using previously acquired immune memory of invasive genetic elements, the CRISPR-Cas9 system is capable of recognizing and destroying specific DNA or RNA sequences, providing an effective barrier against viral infections and horizontal gene transfer^25^.

**Figure 3:**
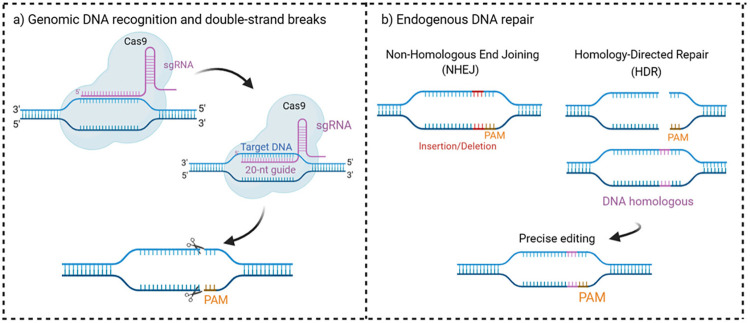
Illustrates the action mechanism of the CRISPR/Cas9 system. (a) shows the target region recognition and the cleavage of the double-stranded DNA based on sgRNA. (b) shows the DNA repair processes known as nonhomologous end-joining (NHEJ) and homology-directed repair (HDR), as well as essential system components such as the single-guide RNA (sgRNA) and the protospacer adjacent motif (PAM). NHEJ is a DNA repair process in which the broken ends of DNA are directly joined together often leading to mutations. In contrast, HDR uses a homologous DNA sequence as a template to repair DNA double-strand breaks with high fidelity.

Since its discovery in 2012, the CRISPR-Cas9 system has been widely recognized as an exceptionally powerful genome editing tool^26^. Its ability to selectively manipulate DNA sequences in a wide variety of organisms, including bacteria, plants, animals, and even humans, represents a significant advancement in biotechnology. By directing a Cas protein to a specific DNA sequence, the CRISPR-Cas9 system can induce precise cuts in the genome, allowing for the insertion, deletion, or modification of genes with unprecedented accuracy (Figure 3).

Compared to other genome editing technologies such as meganucleases, zinc-finger nucleases (ZFNs), and transcription activator-like effector nucleases (TALENs), CRISPR-Cas stands out not only for its efficiency and precision but also for its relative ease of use and affordability^27^. Its fundamental basis lies in the base complementarity between a guide RNA (gRNA) sequence and the genomic target, presenting exceptional versatility in genomic engineering. In addition to its applications in genome editing, the CRISPR-Cas9 system has been explored in a variety of other areas, including molecular diagnostics, gene therapy, pest control, and even data storage^28^.

The native CRISPR-Cas9 systems are classified into two main classes, encompassing six types of Cas effector proteins^29^. CRISPR-Cas9, which belongs to class II and type II, is the most adopted and widely used system in CRISPR-based genome editing strategies. CRISPR-Cas9 typically recognizes a specific sequence called Protospacer Adjacent Motif (PAM), commonly known as NGG (in which N can be any nucleotide), which is essential for its function^30^. In addition to CRISPR-Cas9, other notable Cas proteins include Cas12a, type V, which also recognizes specific PAM sequences, and Cas13, type VI, capable of precisely targeting RNA^31^.

This specific recognition of the sequence by the CRISPR-Cas9 system, such as PAM or NGG for spCas9, is mediated by 17–20 nucleotides at the 5’ end of the guide gRNA^32^. This specificity prevents undesired off-target interactions. The nuclease domains of Cas9, HNH, and RuvC, cleave the target and nontarget DNA strands, respectively. In the absence of a template, the resulting double-strand break is repaired by the nonhomologous end joining pathway, a primary pathway for the repair of DNA double-strand breaks throughout the cell cycle^33^, also during the S and G2 phases, introducing random indel mutations. Alternatively, the homology-directed repair (HDR) pathway uses an exogenous DNA repair template to introduce precise mutations at a specific locus^34^.

### CRISPR-Cas9 and HTLV-1

When exploring the potential of the CRISPR-Cas9 system in the treatment of viral infections, such as HTLV-1, it is crucial to identify strategic genetic targets^19^. Essential for viral replication genes, such as TAX and HBZ, and host-specific genes, can be precisely selected. The HTLV-1 Tax and HBZ proteins play crucial and interconnected roles in viral replication and cellular transformation. As described in previous studies, TAX is a potent transactivator of the viral LTR promoter, driving high-level expression of viral genes^35^. TAX also modulates various cellular signaling pathways, including NF-κB, to promote cell proliferation and survival^36^. In contrast, the HBZ protein acts to counterbalance the effects of TAX, suppressing viral gene expression and promoting viral latency^37^. The dynamic interplay between TAX and HBZ is thought to be critical for establishing the delicate balance between viral replication and host cell transformation that ultimately leads to HTLV-1-associated diseases^38^. Targeting both of these viral regulatory proteins using CRISPR-Cas9 genome editing may therefore be an effective strategy to disrupt this oncogenic process^39^. Suppressing these genes may lead to effective inhibition of viral replication, representing a crucial step towards a functional cure^40^. This precise approach in defining target sequences is vital for successfully guiding therapeutic strategies^41^, offering the prospect of significant advances in the treatment of HTLV-1 infection.

For example, a study conducted by Prawiro *et al*.^42^ provided an illustrative case by identifying a single copy of the hotspot mutation PLCγ1 S345F in an ATLL-derived cell lineage. With CRISPR-Cas9-mediated HDR, they rectified the single nucleotide gene mutation. Remarkably, this restoration to the wild-type PLCγ1 configuration adversely affected crucial functional properties of the ATLL cell lineage, notably its heightened proliferation rate and chemotaxis. Combined with insights gained from inhibiting the PLCγ1 pathway in the ATLL cell lineage, these findings underscore the critical role of mutated PLCγ1 activation in ATLL pathology.

Recently, the vasodilator-stimulated phosphoprotein (VASP) has emerged as a pivotal player in facilitating the efficient transfer of p8 and HTLV-1 Gag, as elucidated by Donhauser *et al*.^43^. The interaction site was pinpointed within the Ena/VASP homology domain 1 (EVH1) of VASP, highlighting its critical role in mediating these viral processes. Using CRISPR-Cas9 methodology, researchers successfully suppressed VASP expression, resulting in a significant decrease in p8 transfer both to the cell surface and to Jurkat T cells. Conversely, despite expectations, the removal of the CCCTC-binding factor (CTCF) did not yield conspicuously visible effects on viral transcription or the examined epigenetic modifications^44^.

In addition, the recent studies by Mohanty *et al*.^45^and Fochi *et al*.^46^ have provided crucial insights into the molecular mechanisms of NF-κB activation and the regulation of viral proteins in HTLV-1 pathogenesis. Mohanty *et al*.^45^ have elucidated the central role of mammalian ubiquitination factor E4B (UBE4B) in NF-κB activation and HTLV-1 TAX polyubiquitination, highlighting its potential as a therapeutic target. In the same study, the influence of UBE4B on K48- and K63-linked polyubiquitination was demonstrated, suggesting its importance in TAX-induced activation of the NF-κB signaling pathway. Moreover, Fochi *et al*.^46^ investigated the interaction of TNF receptor-associated factor 3’ (TRAF3) with HTLV-1 regulatory proteins and discovered its influence on TAX-mediated NF-κB activation. Of note, APH-2 inhibited TAX-dependent NF-κB activation more strongly than HBZ, suggesting distinct roles of viral regulators. Taken together, these results deepen our understanding of HTLV-1 pathogenesis and offer promising opportunities for therapeutic intervention. In addition to the elegant results of the later studies, they also demonstrate the power of CRISPR/Cas9 as a tool for deciphering the crucial viral and host factors involved in HTLV-1 pathogenesis. With targeted genetic modifications, CRISPR/Cas9 offers the opportunity to investigate the specific role of genes and signaling pathways in the development of HTLV-1-associated diseases. These findings can then be incorporated into the development of new therapeutic strategies, making CRISPR/Cas9 an extremely valuable technology in this research field.

In parallel, the recent study by Zotova *et al*.^47^ on the development of monoclonal antibodies (mAbs) have shown promising avenues for targeted therapies against HTLV-1. By characterizing antibodies such as the monoclonal antibody BF4, which is able to stain viral biofilms in HTLV-1-infected T cells, researchers have made significant progress in identifying potential targets for mAb-based interventions. By using genetic engineering methods such as CRISPR-Cas9, researchers have validated these antibodies with limited reactivity, providing a new approach to mAb validation. Identifying BF4 as an anti-CD82 mAb by the genome-wide CRISPR-Cas9 library demonstrates the potential of this approach in spotting therapeutic targets for HTLV-1-associated diseases.

This gene-based approach using CRISPR-Cas9 complements conventional protein- and gene-based methods, offering a promising alternative for mAbs validation and representing a significant step forward in the development of targeted therapies against HTLV-1. Additionally, the use of CRISPR/Cas9 technology can benefit the development of mAbs and vaccines for prophylactic treatments against HTLV-1. CRISPR/Cas9 can enable increased rates of cell line generation, which is crucial for producing and characterizing mAbs. Furthermore, CRISPR/Cas9-based genomic screens can be leveraged to identify the specific epitopes targeted by these mAbs, facilitating a deeper understanding of their action mechanisms.

### HTLV-1 and STLV-1

The human T-cell lymphotropic viruses Type 1 and 2 (HTLV-1 and HTLV-2), along with their simian counterparts (STLV-1 and STLV-2), are part of the primate T-cell lymphotropic virus group (PTLV)^48^. The high genetic similarity between HTLV-1 and STLV-1 strains suggests that most of HTLV-1 subtypes originate from interspecies transmissions between monkeys and humans^1^. Natural infection with STLV-1 leads to the expression of viral proteins such as TAX and SBZ, which are homologous to HTLV-1 proteins HBZ and TAX, respectively, and influence viral signaling pathways LTR and NF-κB^49^.

The application of the CRISPR technique in animal models closely related to humans can be directed to selectively interrupt the expression of these viral proteins. This could allow the investigation of the impact of inhibiting these proteins on the progression of STLV-1 infection, providing valuable information on potential therapeutic strategies in humans^50^. The genetic similarity between HTLV-1 and HTLV-2 subtypes is approximately 60% in nucleic acids and 70% in amino acids, indicating independent evolution from the transmission of Simian T-Cell Lymphotropic Virus (STLV) from non-human primates to humans^43^. The endemicity of HTLV-1 and HTLV-2 in remote populations suggests human infections over thousands of years^51^. In particular, the Efe and Mbuti Pygmies (Congo), considered descendants of proto-African peoples, show infection with both viruses^52^.

The high genetic similarity between HTLV-1 and STLV-1 strains, indicative of interspecies transmission between monkeys and humans^50^, suggests the use of the CRISPR approach in developing specific strategies for these viruses. With CRISPR-Cas9 technology, precise editing of genetic sequences would be possible, allowing modulation of viral gene expression, deactivation of key virus components, or elimination of viral genetic material from infected cells. In the context of STLVs, the CRISPR technique can be employed to understand genetic differences and develop specific intervention strategies aimed at limiting or eliminating interspecies transmission of these viruses to humans.

### Challenges in the precise use of CRISPR

While the results achieved so far are impressive and the use of genome editing (GE) in various living organisms—including important agricultural crops—has become commonplace, it is imperative to consider the inherent challenges in the precise use of the CRISPR-Cas9 system. Issues related to the fidelity of CRISPR-Cas9 systems are constantly addressed, especially concerning off-target mutations^53^. These concerns are of utmost importance due to the potential unintended consequences, emphasizing the need for precise and reliable genetic modifications.

The CRISPR-Cas9 system, although widely used in genome editing due to its effectiveness, is more prone to off-target effects compared to dimeric systems like TALENs and ZFNs, due to the monomeric nature of Cas9^54^. These undesired effects occur when the Cas9 complex binds to unintended regions, triggering cleavage events and potentially resulting in unintended genetic alterations. Strategies are being developed to enhance the specificity of gRNA sequences, aiming to identify and mitigate these potentially problematic regions^55^. To address these challenges, strategies involving modifications in the Cas9 component, adjustments in gRNA, and the use of base editors^56^, such as ribonucleoprotein complexes (RNP), are being explored to ensure more precise and reliable genetic modifications^57^.

Off-target effects in CRISPR-Cas9 systems can be classified into three main types. The first involves regions with other motifs adjacent to the protospacer adjacent motif (PAMs) containing substitutions or mismatches^58^. The second type encompasses regions with PAMs containing insertions or deletions compared to the DNA or gRNA-target spacer, leading to the formation of a small bulge^58^. The third type refers to the cleavage of sequences with different PAM sites. Additionally, two categories of off-target effects by CRISPR are proposed, distinguishing between those expected in genomic regions with high sequence similarity to the target and those unexpected in unrelated genomic regions^58^.

### An overview: recent advances and challenges in CRISPR-Cas9-mediated genome editing for HTLV-1

Recent studies cited here demonstrate the potential of CRISPR-Cas9-mediated genome editing in precisely modifying genetic sequences associated with HTLV-1 pathogenesis. For instance, the correction of specific mutations in ATLL-derived cell lineages with HDR has shown significant adverse effects on cellular functional properties^42-46^. These findings, along with others reported and discussed in the literature, underscore the promise of targeted gene therapy to halt the pathological processes associated with HTLV-1 infection.

However, despite significant advancements, technical and ethical challenges persist in using CRISPR-Cas9 technology to treat HTLV-1 infection. The issue of gRNA sequence specificity and the minimization of off-target effects are areas of intensive focus. As aforementioned, numerous researchers have been dedicated to enhance CRISPR specificity by developing Cas9 enzyme variants with greater selectivity toward the desired target.

Furthermore, screening and validation techniques are essential to identify potential off-targets, thus ensuring the accuracy of genetic editing. Another challenge lies in the efficient delivery of CRISPR technology to target cells, along with the selection of appropriate cell lineages^59^. Moreover, even after the identification of suitable cell lineages, ensuring efficient delivery and expression of the CRISPR-Cas9 system in these cells is crucial, which can be complicated due to cell cultures heterogeneity and transduction difficulty in primary or established cells^59^. This effective delivery is crucial for precisely targeting desired genetic modifications and avoiding unwanted off-target effects, as mentioned earlier.

Genetic therapy requires the precise delivery of the CRISPR-Cas9 system to the specific site where genetic editing is necessary. This process can be especially challenging in hard-to-reach organs such as the brain or heart. Recent advances in delivery technologies, such as viral vectors and nanoparticles, have shown promising results; however, it is necessary to refine such methods to make them sufficiently safe and effective for clinical application^60^. Additionally, it is crucial to ensure that gene editing in human cells is safe and does not induce long-term adverse effects. Clinical trials must be carefully designed and conducted to assess the safety and efficacy of CRISPR therapies before their widespread adoption. Furthermore, ethical considerations, such as safety and equity in access to gene therapy, require continuous attention to ensure the responsible application of CRISPR-Cas9 technology.

## CONCLUSION

As research continues to advance, it is expected that CRISPR-Cas9 technology will play an increasingly important role in the development of targeted therapies for HTLV-1 infection. In the context of intermediate syndrome, the application of CRISPR technology is described as a potentially effective therapeutic approach, regardless of the patient’s clinical stage. Innovative strategies, such as modulation of viral and host-specific genes, have the potential to revolutionize the treatment of conditions associated with HTLV-1, offering new hope for patients affected by this debilitating disease, who currently lack effective therapies.
